# Development of consensus quality indicators for cancer supportive care: a Delphi study and pilot testing

**DOI:** 10.1186/s12913-024-10876-6

**Published:** 2024-03-27

**Authors:** Amelia Hyatt, Karla Gough, Holly Chung, Wendy Wood, Ruth Aston, Jo Cockwill, Spiridoula Galetakis, Meinir Krishnasamy

**Affiliations:** 1 Department of Health Services Research, Peter MacCallum Cancer Centre, Melbourne, Australia; 2 Sir Peter MacCallum Department of Oncology, University of Melbourne, Melbourne, Australia; 3 Department of Nursing, University of Melbourne, Melbourne, Australia; 4 Australian Council on Health Care Standards, Sydney New South, Wales; 5 Assessment and Evaluation Research Centre, Melbourne Graduate School of Education, University of Melbourne, Melbourne, Australia; 6 VCCC Alliance, Melbourne, Australia; 7 Department of Health Victoria, Cancer Support Treatment and Research Unit, Melbourne, Australia

**Keywords:** Cancer supportive care, Oncology, Quality indicators, indicator development, Delphi

## Abstract

**Background:**

High quality supportive care is fundamental to achieve optimal health outcomes for people affected by cancer. Use of quality indicators provides comparative information for monitoring, management, and improvement of care within and across healthcare systems. The aim of this Australian study was to develop and test a minimum viable set of cancer supportive care quality indicators that would be feasible to implement and generate usable data for policy and practice.

**Methods:**

A two-round, modified reactive Delphi process was employed firstto develop the proposed indicators. Participants with expertise in cancer control in Australia, the United Kingdom, and Canada rated their level of agreement on a 7-point Likert scale against criteria assessing the importance, feasibility, and usability of proposed indicators. Relative response frequencies were assessed against pre-specified consensus criteria and a ranking exercise, which delivered the list of proposed indicators. Draft indicators were then presented to a purposive sample of clinicial and health management staff via qualitative interviews at two acute care settings in Melbourne, Australia for feedback regarding feasibility. Desktop audits of online published health service policy and practice descriptions were also conducted at participating acute care settings to confirm health service data availability and feasibility of collection to report against proposed indicators.

**Results:**

Sixteen quality indicators associated with the delivery of quality cancer supportive care in Australian acute healthcare settings met pre-specified criteria for inclusion. Indicators deemed ‘necessary’ were mapped and ranked across five key categories: Screening, Referrals, Data Management, Communication and Training, and Culturally Safe and Accessible Care. Testing confirmed indicators were viewed as feasible by clinical and health management staff, and desktop audits could provide a fast and reasonably effective method to assess general adherence and performance.

**Conclusions:**

The development of quality indicators specific to cancer supportive care provides a strong framework for measurement and monitoring, service improvement, and practice change with the potential to improve health outcomes for people affected by cancer. Evaluation of implementation feasibility of these expert consensus generated quality indicators is recommended.

## Background

The association between cancer supportive care, optimal health outcomes and quality of life is well established [1]. In particular, provision of timely, appropriate, and accessible cancer supportive care can prevent or mediate the impacts of cancer and its treatment across key domains of patient and carer need [2]. In many instances, access to supportive care services to address barriers to care can be a crucial factor underpinning treatment success [3]. Importantly, new conceptual frameworks of cancer supportive care highlight its benefit, underpinning all facets of service delivery across the cancer trajectory [4].

In order to achieve optimal health and quality of life outcomes, cancer supportive care service delivery must be of high quality [5]. However, information regarding the quality of cancer supportive care delivered in acute healthcare centres globally is limited. While the Organisation for Economic Co-operation and Development (OECD) have developed international healthcare quality indicators for benchmarking and comparison between member states inclusive of cancer; no indicators specific to the delivery of supportive care are present [6]. Within Australia, current health performance indicators applied by the Australian Institute of Health and Welfare likewise do not include cancer supportive care [7]. Absence of standardised measurement for monitoring and implementation of supportive care has resulted in variable service availability and quality, both of which are associated with poor outcomes [8].

Measurement of quality through health service performance can enable the development of policy-to-practice guidelines [9], and provide comparative information for monitoring and management both within and across healthcare systems [10]. Standardised metrics provide strong accountability mechanisms for key stakeholders to support improvement in and reduction of low value care [11]. Quality indicators are useful tools to guide measurement of quality and assist healthcare organisations identify areas for performance improvement [12]. In healthcare, the Delphi process is widely used to facilitate quality indicator development [13]. Importantly, however, use of overarching conceptual frameworks are integral in supporting guideline-based quality indicator development [14].

While quality indicators have been developed to facilitate greater standardisation in care delivery, thus overcoming one of the key barriers to quality care [9], their implementation is not always successful. Irrespective of whether cancer-specific or healthcare general quality indicators, a variety of barriers to implementation and use have been identified, ranging from knowledge or behaviour change barriers at the healthcare professional level to leadership and resourcing issues at an organisational level [15, 16]. Identifying the possible barriers or challenges to implementation of quality indicators in a particular context or setting is important to ensure their success as an effective tool to improve quality of care.

The aim of this study, therefore, was to develop a minimum viable set of cancer supportive care quality indicators that would be feasible to implement; generate useful, accurate, and relevant data that appropriately convey the quality of cancer supportive care service delivery at the health service level; target an area of importance where there is a clear gap between current supportive care provision and the level of health outcomes that could be achieved by improvements in the quality of care; and are acceptable to end users, with potential barriers and facilitators to implementation and uptake identified. Results will be used to inform the development of optimal methods for effective implementation of the proposed indicators.

## Methods

### Design summary

This project involved three separate, but interlinked studies. A two-round, modified reactive Delphi process was employed, whereby a scoping review of quality indicators informed the first round, allowing for synthesis of best available evidence and expert opinion while enhancing efficiency of the Delphi process [17–20]. The Delphi technique was selected due to its extensive application in the development of quality indicators in healthcare [13]. After completion of the Delphi study, newly developed quality indicators for cancer supportive care were then assessed for feasibility through semi-structured interviews and comprehensive desktop audits at two metropolitan healthcare services in Australia. To best facilitate ease of understanding, methods for each sub-study have been outlined separately.

This study was reviewed and approved by the University of Melbourne HREC (approval no: 1955021.1) and is part of a larger suite of projects inclusive of other Delphi studies conducted concurrently to gain expert consensus on various issues associated with cancer supportive care [4].

### Conceptual framework

A framework of cancer supportive care, developed by our group was used as the underpinning framework to guide quality indicator development in addition to the findings from the multi-round Delphi process [4].

#### Advisory group

An advisory group of seven national stakeholders in cancer supportive care was established to provide oversight and guidance across all project operations. Members comprised policy makers, senior academics, non-government cancer organisation leaders, and consumer advocates.

#### Participating healthcare services

Two acute care settings located in Melbourne, Australia were selected for quality indicator feasibility testing. Selection was made due to comparative differences between site case-mix, particularly with regard to typical social and demographic characteristics of attending patients. Specifically, one site had a large catchment area in a socially disadvantaged area of Melbourne, within a large culturally and linguistically diverse community. Resourcing in this context is challenging, and there are limited external supportive care services available for referral. The second site was in a relatively affluent community, had adquate resourcing available, and established cancer supportive care plan. It was anticipated that these differences would allow for assessment of feasibility of the quality indicators across diverse settings.

## Quality Indicator development

### Delphi survey

A two-step scoping review was undertaken to develop a list of potential structural and process quality indicators to present for expert review in the first round of the Delphi. Use of both structural and process indicators for quality measurement is advantageous, as associated data are often routinely available, making them easy to measure and interpret [21].

The first step included a comprehensive review of published qualitative, quantitative, and mixed methods papers, as well as systematic, scoping, and rapid review journal articles reporting on the provision of cancer supportive care in acute clinical contexts. Papers were identified through searches on Pub Med, Ovid MEDLINE, CINAHL, PsycINFO, and Cochrane between 21st March 2019 and 23rd May 2019. Search terms and variations of terms were deliberately broad to address the scope of supportive care and included: “psychosocial support”, “social support” “spirituality”, “palliative care”, “needs assessment”, “quality of health care”, “quality indicators”, “structural indicators”, “process indicators”, “outcome indicators”, “physical needs”, “psychological needs”, “social needs”, “information needs” and, “spiritual needs”. The Boolean operators of AND/OR were used, as was backward and forward citation chasing.

Second, a review of publications identified through websites of leading international cancer organisations and government departments that presented health policy and national and international guideline documents on implementation and quality of supportive care in cancer was conducted. Healthcare safety and quality frameworks of countries leading supportive care efforts including Australia, the United Kingdom, Canada, and America were also included.

Papers, guidelines, and frameworks published in English from 2000 to 2019 were included to ensure seminal papers in the field were identified. Two independent assessors reviewed abstracts and relevant full citations to develop an initial list of 61 potential quality indicators. As part of this process, indicators were also deliniated into categories informed deductively via published guidance materials. The refreshed cancer supportive care conceptual framework was utilised to support this identification, synthesis and integration of guidance material into indicators and categories. Duplicate or overlapping indicators and outcome indicators reliant on multiple data points or medical record systems to access quality data, which were deemed impractical to use in practice, were removed [21]. A final list of 48 indicators was available for inclusion in the initial round of the Delphi process.

### Participants

Criteria used to select potential participants for the Delphi panel included experience developing, advising on, delivering, or receiving supportive care in cancer. Potential participants working in clinical, research, policy, quality, and cancer consumer advocacy roles in Australia, the United Kingdom, and Canada (countries recognised as leaders in cancer supportive care) were identified by the operational group and invited to participate. Specific consideration was given to the inclusion of participants working in specialised areas of cancer supportive care, such as people who suppport or provide care to culturally and linguistically diverse and Indigenous cancer consumers. Participants were encouraged to forward the study invitation to colleagues whom they felt would also be appropriate to participate in the Delphi component according to study criteria (snowball recruitment).

### Procedure

Invitations for experts identified by the advisory group and through snowball recruitment were sent via email, with interested individuals directed to complete an online consent form. Participation was described as completing both Delphi rounds. The first Delphi round was open between 21/09/20 to 13/10/2020, and the second from 23/11/20 to 8/12/2020. Two reminder emails were sent for each round to optimise participation. Both surveys included clear descriptions regarding Delphi development, aims, and purpose. Consent forms and Delphi surveys were delivered via a REDCap electronic data capture tool [22].

#### Round 1: importance, feasibility, and usability of proposed quality indicators

Three criteria—importance, feasibility, and usability—were used to establish expert consensus on quality indicators consistent with published guidelines [23]. These were assessed using the following questions:

**Importance:** Will this indicator target an area of importance where there is clear gap between the actual and potential level of healthcare that can be influenced by improvements in the quality of care?

**Feasibility:** Will data required to use this indicator be feasible to access, readily available at all hospitals, and will the time and cost to access and analyse be reasonable?

**Usability:** Will the intended audience (policy makers, clinicians, and consumers) be able to understand the results generated via this indicator?

Each question comprised a 7-point Likert item, with participants rating their level of agreement from ‘1’ (*strongly disagree*) to ‘7’ (*strongly agree*).

#### Round 2: ranking consensus indicators

Participants were provided with a randomly ordered list of quality indicators deemed ‘necessary’ (analysis method described below) based on results from the first Delphi round and asked to rank these in ascending order from the most to least useful, important, and feasible.

Participants were also asked to respond to three statements regarding the indicators as a set using the same response options described above:

1. Are there any indicators listed above which are routinely collected as part of hospital accreditation or national standards that you are aware of?
2. Do you believe that any of the indicators listed could be removed?
3. Do you think that any indicators are missing?

Corresponding open text sections provided participants with the opportunity to elaborate on their response to each statement.

### Data analysis

All data analysis was performed in Excel. For the first Delphi round, responses to Likert items were recoded to discrete variables comprising two categories: highly rated (*agree* or *strongly agree*) or not highly rated (*somewhat agree* through *strongly disagree*); then relative frequencies were calculated for each variable. Next, relative frequencies were assessed against strict pre-specified consensus criteria to ensure a minimum viable set of quality indicators for cancer supportive care: ‘necessary’, at least 75% of experts *agreed* or *strongly agreed* with inclusion of the quality indicator against all three selection criteria; and ‘supplementary’ (60–74%) or ‘unnecessary’ (< 60%), less than 75% of experts *agreed* or *strongly agreed* with inclusion of the quality indicator against all three selection criteria.

For the second Delphi round, medians and interquartile ranges were calculated based on expert rankings of quality indicators deemed ‘necessary’ in the first round, with lower median scores indicating a higher overall ranking. Responses to items regarding the indicators as a set were recoded into discrete variables comprising three categories: no (*disagree*, *strongly disagree*, *disagree*, and *somewhat disagree*); unsure (*neither agree or disagree*); and yes (*strongly agree*, *agree*, and *somewhat agree*); then frequencies were calculated for each variable. Open text responses were analysed using qualitative content analysis [24].

## Quality indicator testing

### Qualitative interviews

#### Participants

Criteria used to select relevant participants for quality indicator review via qualitative interviews comprised: working as an oncologists, cancer nurses, and allied health professionals, as well as senior health service management who were involved in policy, strategy, and clinical implementation of cancer supportive care, and were located at the two participating health services. Potential participants were identified purposively by the principal investigator at each hospital site and approached via email, which included study information and a link to an online consent form hosted on REDcap^25^.

#### Procedure

Healthcare professionals and managers interested in participating were directed to complete the online consent form, after which the study research assistant contacted them via email to arrange an interview and share a copy of the quality indicators. Due to COVID-19 pandemic restrictions interviews were conducted via video conferencing (Zoom) or telephone, depending on the participant’s preference.

Interviews aimed to elicit expert feedback regarding the feasibility of implementing the quality indicators into acute healthcare settings and generate further understanding regarding the barriers and facilitators to implementation and delivery of quality cancer supportive care (as describe by the quality indicators). Semi-structured qualitative interview schedules developed specifically for this study (supplementary file 1)comprised the following four questions:

1. What policies and/or practices and/or services that address these indicators already exist within the health service?
2. Do you find the proposed quality indicators feasible or acceptable to implement?
3. Would use of the proposed quality indicators generate useful data for measuring quality supportive care?
4. What would facilitate implementation of these quality indicators into practice?

### Data analysis

Interviews were audio-recorded and transcribed verbatim, and transcripts stored on a secure server and imported into NVivo 12 for analysis using interpretive description [25]. Data were reviewed predominantly deductively against research objectives (whether quality indicators were feasible, acceptable, and useful); however, an inductive lens^28^ was applied to suggestions regarding effective implementation and suggested improvements. A member of the research team experienced in qualitative analysis conducted all data analysis (AH); with a further 20% of interviews re-analysed by another member of the research team (HC). Discrepancies in codes and categories between the two reviewers were reviewed and discussed until consensus was achieved, then themes were developed and finalised.

### Desktop audit

#### Participants

Desktop audits were conducted concurrently with the interviews (HC) to identify whether data regarding the quality indicators could be feasibly obtained via this mechanism. Desktop audits (also known as ‘desk review’) are commonly used in the global health, humanitarian and environmental sectors, and offer a pragmatic approach to overview relevant documents on a given topic, identify gaps in evidence, and justify primary research or inform questions to be explored through primary research [26, 27].

Two public acute care settings located in Melbourne, Australia (described above) were purposively selected for the study.

#### Procedure

Publicly accessible policy, strategy, and informational documents for each site were identified and assessed to determine whether relevant organisational policies and practices were present to measure performance against each quality indicator. A customised desktop audit data extraction spreadsheet was developed to facilitate data extraction. This tool is available from the authors. Key policy documents were assessed to determine whether they referenced any policy, practice, or process which could be interpreted as providing support for meeting any of the 16 proposed quality indicators. Relevant data were extracted and recorded per hospital site for each quality indicator. For both hospitals, alignment between data sources (desktop audit and interviews) is summarised, and referred to below as the proportion of concordance.

#### Data analysis

Data collected using the desktop audit tool for each site against each quality indicator were assessed as being:

**Present:** Enough data were present to establish whether the quality indicator was met.

**Absent:** Insufficient data were present to establish whether the quality indicator was met.

A member of the research team (HC) completed the audit for both sites. A second reviewer (AH) then re-assessed all evidence and decisions outlined in the audit tool. Examples of evidence of presence and absence were discussed by the study team and agreed upon by consensus. Data from the desktop audit were then compared with data from participant interviews describing actual practice to determine concordance. Results were tabulated to showcase the validity of using a desktop audit to assess a hospital’s implementation of cancer supportive care as described by the quality indicators.

## Results

### Delphi panel

As noted above, this study was part of a larger suite of research projects exploring aspects of cancer supportive care. A total of 83 eligible individuals were identified, 56 directly by the research team and a further 27 through snowball recruitment. Of these, 70 participants consented to take part in the broader project, with a smaller subset of participants taking part in this Delphi study, 35 in the first round, and 34 in the second (see Table 1).

**Table 1:** Characteristics of participants in Delphi rounds one and two and qualitative interviews

Characteristic	Delphi Survey				Qualitative Interview			
	Round one		Round two		Site A		Site B	
	*n* = 35		*n* = 34		*n* = 5		*n* = 6	
	n	%	n	%	n	%	n	%
**Yrs worked in supportive care role**								
Mean, standard deviation	16	12	15	12	15	10	16	11
Minimum, maximum	2	45	2	45	6	37	6	37
**Role Category**								
Clinician	14	41	13	39				
Researcher	8	24	6	18				
Policy representative	3	9	2	6				
Quality representative	2	6	4	12				
Consumer representative	4	12	5	15				
Carer	1	3	2	6				
Other	2	6	1	3				
Missing	[1]		[1]					
**Gender**								
Male	5	15	5	15	2	40	2	33
Female	28	85	28	85	3	60	4	67
Missing	[2]		[1]					
**Location**								
Australia	30	88	28	85				
International	4	12	5	15				
Missing	[5]		[28]					`
**Stakeholder Category**								
Health Professional					4	80	4	67
Health Service Manager					1	20	2	33
**Clinician Category**								
Doctor					1	20	4	66
Nurse					3	60	1	17
Allied Health					1	20	1	17

### Delphi round 1

Of the 48 proposed quality indicators, a total of 16 indicators met the pre-specified consensus criteria of ‘necessary’; that is, at least 75% of experts *agreed* or *strongly agreed* that the indicator was important, usable, and feasible (see Table 2). Indicators that formed the minimum viable set were categorised as follows: Communication and Training (*n* = 4), Screening (*n* = 3), Data Management (*n* = 2), Referral (*n* = 3), and Culturally Safe and Accessible Supportive (*n* = 4). No indicators were retained from the Governance or Policy categories.

**Table 2:** Percentage of participants who agreed or strongly agreed with the inclusion of each indicator against all three selection criteria (*n* = 35)

Category/Indicator		% highly rated^a^			Selection criteria meeting threshold^b^
		Importance	Feasibility	Usability	
Governance					
1	This organisation has a dedicated supportive care committee	77	71	69	I
2	The organisation documents requirement for establishment or existence of a supportive care committee that articulates to one of the National Standards quality committees	74	69	69	
3	The organisation has a senior (executive) role identified as the organisation supportive care champion	80	71	74	I
Policy					
4	The organisation has an accessible Supportive Care policy	80	77	66	I, F
5	The organisation has a Supportive Care Policy that is current (updated every 12 months)	71	69	63	
6	The organisation has a Supportive Care Policy that that describes a framework for the provision of supportive care	86	71	77	I, U
7	The organisation has a Supportive Care Policy that directs supportive care reporting within a dedicated organisational reporting framework	86	80	74	I, F
8	The organisation has Supportive Care Policy that directs specific reporting metrics	80	63	71	I
9	The organisation has Supportive Care Policy that directs specific patient experience reporting requirements	89	66	74	I
10	The organisation has Supportive Care Policy that documents reporting responsibility for supportive care data to a government agency (if required)	63	63	51	
11	The organisation has Supportive Care Policy that documents reporting requirements a relevant organisation executive committee (e.g. a hospital board)	74	69	69	
12	The organisation has Supportive Care Policy that documents reporting requirements to their Executive Quality and Safety Committee	80	71	71	I
13	The organisation has Supportive Care Policy that documents the role of consumers in the design of supportive care programs evaluation and reporting	86	69	69	I
Communication and Training					
14	The organisation has formal processes in place to guide information-sharing, discussion, and education about supportive care available for staff, patients and family carers	94	89	86	I, F, U
15	The organisation has a documented process that requires relevant staff undertake supportive care training (e.g. the eviQ modules)	91	77	80	I, F, U
16	The organisation has a documented process to ensure staff training for supportive care is recorded	77	80	71	I, F
17	The organisation has a documented process to ensure individuals have opportunity for discussion of their supportive care needs at any stage along their illness or treatment continuum	94	80	83	I, F, U
18	The organisation has a documented process to ensure that patients and families understand what supportive care is (e.g. the WeCan resources)	83	71	71	I
19	The organisation has a documented process that sets an expectation that patients and families feel able to ask about supportive care needs	91	80	80	I, F, U
20	The organisation has availability of resources to support carers and family members	97	71	89	I, U
21	The organisation has a dedicated facility or space to address wellbeing of patients, carers and family members who attend the hospital (e.g. a wellbeing centre)	74	54	63	
Screening					
22	The organisation undertakes supportive care screening	91	83	80	I, F, U
23	The organisation has a documented process that sets out what supportive care screening tool should be used for all patients across the organisation	89	86	80	I, F, U
24	The organisation has nominated person(s) to undertake Supportive Care screening	83	63	71	I
25	The organisation has a documented process to inform when and how often supportive care needs screening should be undertaken	91	71	71	I
26	The organisation has a documented process for how supportive care data are collected (face to face/electronic)	83	80	77	I, F, U
Data Management					
27	The organisation The organisation has a documented process for how supportive care data are to be used in clinical consultations	83	66	69	I
28	The organisation has a documented process for how supportive care data are to be stored	83	74	71	I
29	The organisation has a documented process for how supportive care data are to be used for research purposes	83	74	77	I, U
30	The organisation has a documented process for how supportive care data are to be used to identify patients at risk of high unmet need	94	80	83	I, F, U
31	The organisation has a documented process for how supportive care information is recorded in the patient’s medical record	94	83	89	I, F, U
Referral					
32	The organisation has processes in place for referring patients to access supportive care services if a need is identified	97	83	86	I, F, U
33	The organisation has a documented process to ensure that supportive care needs are asked about and considered as part of a multidisciplinary care team meetings	91	74	77	I, U
34	The organisation has a documented process for internal referral of patients for unmet needs	94	83	83	I, F, U
35	The organisation has a documented process for external referral of patients for unmet needs	91	69	80	I, U
36	The organisation has a documented process for referral of patients for unmet needs based on risk stratification	80	57	71	I
37	The organisation has a documented process for recording referrals made	97	80	86	I, F, U
38	The organisation has a documented process for recording referrals taken up by patients	74	57	63	
39	The organisation has a documented process for linking uptake of referrals to relevant health outcomes	83	43	54	I
40	The organisation has a documented process for encouraging cross sector referrals to ensure patients have access to the services they need irrespective of organisation-specific resource	97	63	74	I
Culturally Safe and Accessible Supportive Care					
41	The organisation is committed to providing culturally safe and accessible care for all Australians	94	71	71	I
42	The organisation has a documented process to ensure individuals with special needs are catered for	89	74	74	I
43	The organisation has a documented process to ensure cultural sensitivity	94	80	86	I, F, U
44	The organisation has a documented process to ensure interpreters are available if needed	94	80	86	I, F, U
45	The organisation has a documented process to ensure information is available in other languages or in different format for low literacy readers	91	77	83	I, F, U
46	The organisation has an Aboriginal and Torres Strait Islander patient liaison officer	86	69	80	I, U
47	The organisation has a Reconciliation Action Plan	86	74	74	I
48	The organisation has cultural competency training available for all staff	94	89	89	I, F, U

^a^Cells with relative frequencies ≥75% are coloured green; cells with relative frequencies between 60 and 74% are coloured yellow; and cells with relative frequencies less than 60% are not coloured

^b^*I* Importance: *F* Feasibility: *U* Usability

Notably, 42 of 48 candidate indicators met pre-specified consensus criteria for importance. Of these 42 indicators, three met criteria for feasibility but not usability, six met criteria for usability but not feasibility, and 17 did not meet consensus criteria for feasibility or usability. All six indicators that did not meet consensus criteria for importance, were also deemed unfeasible and unusable. (Table 2).

### Delphi round 2

Results from the indicator ranking exercise are presented in Table 3. The top five ranked indicators came from the Communication and Training, Screening, and Referral categories.

**Table 3:** Median rank and interquartile range for the minimum viable set of quality indicators for cancer supportive care (n = 34)

Quality Indicator	Category	Median rank (IQR)^a^
The organisation has a documented process to ensure individuals have opportunity for discussion of their supportive care needs at any stage along their illness or treatment continuum	Communication and Training	3 (1, 6)
The organisation undertakes supportive care screening	Screening	3 (1, 7)
The organisation has processes in place for referring patients to access supportive care services if a need is identified	Referral	5 (2, 10)
The organisation has a documented process that sets out what supportive care screening tool should be used for all patients across the organisation	Screening	6 (4, 11)
The organisation has formal processes in place to guide information-sharing, discussion, and education about supportive care available for staff, patients, and family carers	Communication and Training	7 (3, 10)
The organisation has a documented process that sets an expectation that patients and families feel able to ask about supportive care needs	Communication and Training	8 (3, 12)
The organisation has a documented process for internal referral of patients for unmet needs	Referral	8 (4, 13)
The organisation has a documented process for how supportive care data are to be used to identify patients at risk of high unmet need	Data Management	9 (5, 12)
The organisation has a documented process for how supportive care information is recorded in the patient’s medical record	Data Management	9 (7, 12)
The organisation has cultural competency training available for all staff	Culturally Safe and Accessible Supportive Care	10 (8, 14)
The organisation has a documented process to ensure information is available in other languages or in different format for low literacy readers	Culturally Safe and Accessible Supportive Care	10 (8, 14)
The organisation has a documented process to ensure cultural sensitivity	Culturally Safe and Accessible Supportive Care	11 (6, 13)
The organisation has a documented process for recording referrals made	Referral	11 (7, 13)
The organisation has a documented process to ensure interpreters are available if needed	Culturally Safe and Accessible Supportive Care	11 (7, 14)
The organisation has a documented process that requires relevant staff undertake supportive care training	Communication and Training	11 (8, 14)
The organisation has a documented process for how supportive care data are collected (face to face/electronic)	Screening	12 (8, 15)

^a^*IQR* interquartile range

Most participants (30 of 34) were unsure whether any hospital- or national-level indicators were currently routinely collected; however, five participants suggested some crossover between the indicator set and existing hospital accreditation or national standards [29]. Cancer supportive care screening was specifically highlighted as a possible ‘crossover’ indicator.

Approximately three-fifths believed that no indicators could be removed and no indicators were missing (*n* = 21 and *n* = 20, respectively). Seven participants whose responses suggested they believed some/any of the indicators could be removed provided comments in the open text section. These comments clarified that experts did not believe any indicators should be removed, but rather that some indicators could be collapsed or integrated to remove any perceived duplication. For example, the process of making and documenting referrals could be combined; or a documented process for organisational cultural sensitivity could be combined with cultural competency training. These responses were noted for future action by the project team but not addressed as part of this study as were outside of the scope of this work.

Five participants who believed that some/any indicators were missing elaborated on their response in the open text section. Again experts did not feel that entire indicators were missing, rather they made suggestions about information which could be added, such as adding ‘re-screening’ to the screening indicator to emphasis the iterative nature of this task and the importance of understanding patients’ supportive care needs at different timepoints.

### Qualitative interviews

A total of 11 healthcare professionals and health service managers participated across both health services (n = 5 and n = 6 respectively)(Table 1). Participants at one site were predominantly doctors (n = 5, 66%,), and at the other were nurses (n = 3, 60%). Average length of time working in supportive care were similar across sites, with 15 years (sd = 10) at site a, and 16 years at site b (sd = 11).

Stakeholders provided considerable insight into the usefulness, appropriateness, and feasibility of the quality indicators. Table 4 details stakeholder feedback across all indicator groupings, according to five key themes: Feasible, appropriate, and useful; Investment is prioritised; An integrated approach is needed; System alignment is essential; and Cultural safety and inclusive care.

**Table 4:** Qualitative interviews: clinician key themes

Theme	Insights from the data	Exemplar
**Feasible, appropriate, and useful**	Overall, health professionals felt that the list of 16 Quality Indicators cover all key areas pertaining to cancer supportive care. Clarifying responsibility for undertaking supportive care activities will help ensure indicators can be implemented and result in tangible action.	*“I think you have actually covered everything and they’re all quite valuable in its own right” Site A, Health Professional, 02*
**Investment is prioritised**	Implementation of the indicators requires adequate investment, resourcing and staffing to be feasible. Current supportive care needs screening is hampered by lack of adequate funding, undermining ability of healthcare. Professionals to meet optimal requirements. Health professionals lack adequate time for meaningful conversations with patients at regular intervals throughout their cancer trajectory. Funding liaison nurses or nurse consultants is a priority to help address this deficit. Supportive care training and education is an area for funding to ensure indicators are feasible to implement.	*“And it needs to have allocated funds around that, we’re funded in health services for activity, you know… giving chemotherapy, but we’re not funded for supportive care that’s sort of supposed to be all bundled up in one package” Site A, Health Professional, 01.*
**An integrated approach is needed**	Supportive care indicators must be implemented in the context of comprehensive, integrated care delivery. Patients need to understand the reason for a referral and how that service can assist them in the broader context of their care, to prevent patients declining referrals. A minimum but comprehensive set of services available for referral must be specified and there needs to be adequate resource to deliver them.	*“Sometimes people think: ‘oh I’m not ready for palliative care not understanding what we can offer’. So I think maybe sometimes patients refuse, when if it had been explained properly maybe they wouldn’t. ‘Cause a lot of people think palliative care is end of life where it’s so much more than that.” Site B, Health Professional, 05.*
**System alignment is essential**	System limitations impede regular repeat screening for patients across their experience of care. Implementing screening quality indicators wil help overcome this if specified as part of documented processes. In the absence of standardised approaches different referral processes between and within different units at both health services have emerged. Consequently, clinicians need to operate outside of standard protocols to ensure that people have access to the services they need. When there is lack of access to in-house services or knowledge about external services available, clinical staff may avoid completing referrals altogether. Having quality indicator documented and standardised approaches for identifying, making, processing and recording internal and external referrals is important to enable supportive care in acute health services. Locating screening data within complex health records is problematic. For example, when scanning completed screening tools into the health record is part of a documented process, finding these tools becomes challenging. Policies documenting standardised data storage and usage will be important for effective implementation of the quality indicators.	*“Screening might be done – there’s a form that can get filled out that gets filed in the record, but it’s not acted on because there’s then no services to refer on to” – Site B, Health Profesional, 03.* *“So if someone does need physio we actually have to tell them to go to their GP and get a referral from their GP. Or if they really desperately need it we actually have to admit them to the hospital to get them reviewed” Site B, Health Professional, 04.* *“When it gets scanned in at the moment in the EMR, unless you know what date the tool was done on to look for where in the episode it would’ve scanned in they’re almost impossible to find. It doesn’t have like a dedicated place of where it goes to, it just gets scanned in on whatever day the label is stuck on the form... So if you put the wrong label on it could go anywhere. [laughs] -another reason why nobody looks at it ‘cause they can’t find it.” Site B, Health Professional, 04.*
**Cultural Safety and inclusive care**	Both health services had mandatory and documented processes associated with cultural competency, cultural sensitivity, and interpreter services. However, neither had a documented or formal processes for ensuring that information, or screening tools were available in languages other than English. Where screening tools are only available in a few languages they are inadequate to deliver quality supportive care. Involving interpreters in supportive care screening can be challenging due to the time required and complexity of organising interpreter availability to meet clinical appointment times.	*“The distress thermometer’s not translated so, you know, there’s that definite barrier to people actually filling it in and then supportive care being indicated for referrals to be generated and follow up to occur” Site B, Health Professional, 08.*

Importantly, in both hospitals, cancer supportive care screening was predominantly completed by nurses working in the chemotherapy day units. Consequently, participants perceived that generally supportive care training and education was seen as ‘relevant only to nurses’ rather than all healthcare professionals. This had significant impacts on the quality of cancer supportive care provided, with some respondents describing this as highly clinician dependent whether patients were able to receive the help they needed. A more comprehensive definition specifying that all health professionals are responsible for the delivery of supportive care, as outlined in the quality indicators, was suggested.

*“I think sometimes it’s clinician dependent, so whether or not whoever the health professional that the patient is seeing is aware of what supportive care is available or if they prioritize that. So… sometimes they’re well informed and then sometimes they have no idea that there’s other supports beyond their doctor’s appointments and their treatment appointments”* Site A, HP8.

### Desktop audit

A total of 10 publicly and/or internally available published materials documenting relevant organisational policies and practices available across each participating hospital were identified. Desktop auditing to determine cancer supportive care performance against the proposed quality indicators delivered mixed results when compared with stakeholder data describing actual practice (Table 5).

**Table 5:** Desktop audit and stakeholder interview data. Hospital performance against the 16 proposed quality indicators

Hospital	Site A: Information Present (✔) or Absent (X)		Site B Information Present (✔) or Absent (X)	
Indicator	Desktop Audit:	Stakeholder Interview:	Desktop Audit:	Stakeholder Interview:
1. The organisation undertakes supportive care screening	✔	X	✔	✔
2. The organisation has a documented process that sets out what supportive care screening tool should be used for all patients across the organisation	X	X	✔	✔
3. The organisation has a documented process for how supportive care data are collected (face to face/electronic)	X	✔	X	✔
4. The organisation has processes in place for referring patients to access supportive care services if a need is identified	✔	X	✔	✔
5. The organisation has a documented process for internal referral of patients for unmet needs	X	X	✔	✔
6. The organisation has a documented process for recording referrals made	X	X	✔	✔
7. The organisation has a documented process for how supportive care data are to be used to identify patients at risk of high unmet need	X	X	✔	✔
8. The organisation has a documented process for how supportive care information is recorded in the patient’s medical record	X	✔	✔	✔
9. The organisation has a documented process to ensure individuals have opportunity for discussion of their supportive care needs at any stage along their illness or treatment continuum	X	X	✔	✔
10. The organisation has a documented process that sets an expectation that patients and families feel able to ask about supportive care needs	X	X	X	X
11. The organisation has formal processes in place to guide information-sharing, discussion, and education about supportive care available for staff, patients and family carers	X	X	✔	✔
12. The organisation has a documented process that requires relevant staff undertake supportive care training (e.g. the eviQ modules)	X	X	✔	✔
13. The organisation has cultural competency training available for all staff	✔	✔	✔	✔
14. The organisation has a documented process to ensure cultural sensitivity	✔	✔	✔	✔
15. The organisation has a documented process to ensure interpreters are available if needed	✔	✔	✔	✔
16. The organisation has a documented process to ensure information is available in other languages or in different format for low literacy readers	✔	X	X	X

For the hospital that had developed comprehensive policy documents pertaining to cancer supportive care (Site B), the desktop audit provided a 94% accurate method to assess performance. However, for the other hospital, which did not have overarching supportive care policy, it was more difficult to determine health system performance using this method, as activities and pockets of excellence developed organically through individual champions or specific services (25% concordance). However, despite these challenges, overall across both hospitals, the desktop audit did accurately determine performance 69% of the time.

Importantly, when considering the feasibility and usefulness of the indicators to be implemented in practice, desktop audits may provide a pragmatic method to assess general adherence and performance.

## Discussion

This Delphi study has delivered a comprehensive list of 16 quality indicators associated with the delivery of quality cancer supportive care in Australian acute care hospitals, providing a framework for measurement and monitoring, service improvement, and practice change. Indicators deemed ‘necessary’ mapped to five key categories: Screening, Referrals, Data Management, Communication and Training, and Culturally Safe and Accessible Care. Importantly, the categories and related indicators selected comprehensively cover a range of mechanisms by which key aspects of cancer supportive care can be effectively delivered and maintained. This is illustrated by the fact that quality indicators which focused on workforce capacity building (e.g., communication training) and infrastructure (e.g., data management) were deemed equally necessary as those which focused on the delivery of care (e.g. completion of screening or referrals). Having appropriate and functional systems to deliver care and capture data, as well as a skilled workforce, are integral components of quality care delivery in any healthcare context [30].

Based on expert ratings, almost all candidate indicators (42 of 48) met consensus criteria for importance. Perceptions of importance related to the extent to which monitoring care components associated with an indicator enabled assessment or identification of variability in care, and that adoption of associated indicators would contribute towards standardising the provision of supportive care and, hence, improve the quality of its provision [23]. However, the feasibility of data collection and usability of resulting information proved to be critical factors in determining whether indicators were retained or not. Approximately half (22 of 42) of the candidate indicators that met consensus criteria for importance, fell short of those same criteria for feasibility. Ensuring that requisite data can be collected with minimal effort within the normal flow of clinical care underpins the criteria of feasibility for quality indicators, and facilitates different levels of data collection, use and reporting [23]. The careful consideration by experts regarding key factors associated with implementation and use of the cancer supportive care quality indicators is a particularly important component of our work and bodes well for their utility.

The exclusion of governance and policy indicators among those indicators prioritised through the Delphi process is important to note. It may be that experts in our study questioned the efficacy of policy frameworks in facilitating implementation or practice change in cancer supportive care. Indeed, literature has consistently highlighted the failure of policy alone to successfully drive practice change in oncology and other chronic disease settings [31, 32] due to a lack of associated processes to guide implementation and availability of adequate resourcing [31, 32]. However, examination of the final quality indicator set suggests that each indicator is underpinned by a formal process or articulated approach to its use or reporting, and that there is a formalised set of policies or procedures present within an organisation to guide practice, monitoring, and reporting; in other words, a governance process. By excluding the more formalised governance or policy indicators, participants may have been endorsing a system-level approach to integration of supportive care as a fundamental component of quality cancer care delivery, albeit underpinned by policy imperatives [4].

Overall, pilot testing the proposed quality indicators across two hospitals demonstrated their feasibility. However, clinical staff identified key issues that need to be addressed to ensure successful implementation. Namely, systemic barriers, funding gaps, and siloed workflows that currently undermine service provision associated with screening and delivery of supportive care. Importantly, activities associated with the proposed quality indicators such as supportive care screening, referrals, and data collection were identified as being especially difficult to integrate into existing processes and systems because of resource constraints, limiting their applicability and effectiveness. Therefore, investment in resourcing (workforce and process issues) were proposed as necessary components to optimal and effective quality indicator implementation [16]. Implementation of the proposed quality indicators requires attention to appropriate funding to achieve performance improvement. It is important to note that evidence from work by our group indicates that investment in quality cancer supportive care can confer significant social return on investment at both the patient- and system-levels [33].

Motivation to improve care is an important facilitator to successful implementation of quality indicators [16, 34]. Health professionals in this study discussed how the quality indicators could assist them to improve care, overcoming systematic issues and barriers to delivery of cancer supportive care. Gathering data directly from health professionals and comparing these against a desktop audit allowed us to establish whether data associated with quality indicators was readily available, and accurately reflected the quality of care provided. Findings tentatively support the use of desktop audits to establish health service performance against proposed quality indicators; however, further assessment may be required as the quality of documents or reports available; their recency and context may impact their value.

Equal access to healthcare and associated benefits and outcomes is a core tenant of patient-centred and value-based healthcare [35, 36]. While Australia appears on many international metrics as a leader in healthcare quality [37], Indigenous and culturally and linguistically diverse Australians still face significant disparities in healthcare access and outcomes [38, 39]. Current national quality indicators employed by the Australian Institute of Health and Welfare as part of the Health Performance Framework do not specifically assess or target health system performance activities designed to ameliorate inequities [7]. Our proposed indicators specifically measure essential processes, programs, and activity associated with equitable access to cancer supportive care. Inclusion of these indicators into national monitoring programs offers a novel and important opportunity to determine the quality of cancer supportive care provision through a value-based healthcare lens, with an overt focus on delivery of equitable care.

### Limitations

While a large range of experts in oncology participated in the Delphi process, a higher proportion of consumer and carer advocates would have been beneficial, along with those providing care to priority populations including culturally and linguistically diverse, first nations, people with a disability, and others. Further, while every effort was made to encourage international participation via purposive email selection and snowball methods, only a small number agreed to participate. Additionally, feasibility testing only occurred within two metropolitan healthcare services in Australia, both located within the same city. Our results therefore may not be generalisable to other healthcare settings such as: rural or remote healthcare services, Aboriginal community controlled healthcare organisations, or other sub-acute or community care settings.

In terms of implementation, it will be important to examine whether the quality indicators are feasible in a real-world setting. Equally, the small number of expert comments regarding the combination or additions to the proposed indicators should be further explored, specifically in terms of how these suggested changes may facilitate or impede implementation. It is recommended that the quality indicators proceed to an assessment of clinical utility and evaluation regarding the feasibility of implementation.

## Conclusion

Cancer supportive care is essential for the delivery of optimal cancer care and health outcomes. However, without access to a quality framework that can inform the implementation of supportive care and focus evaluation on consensus quality criteria, the provision of effective supportive care will remain variable. The development of 16 quality indicators specific to cancer supportive care makes an important contribution to improving health system and service quality and efficiency, and health outcomes for people affected by cancer. Evaluation of implementation feasibility of these expert, consensus generated quality indicators is recommended.

## Data Availability

The datasets generated during the current study are available from the corresponding author on reasonable request and HREC approval conditions.

## Abbreviations

OECD: Organisation for Economic Co-operation and Development
HREC: Human Research Ethics Committee
